# Identifying Adult Dengue Patients at Low Risk for Clinically Significant Bleeding

**DOI:** 10.1371/journal.pone.0148579

**Published:** 2016-02-05

**Authors:** Joshua G. X. Wong, Tun Linn Thein, Yee-Sin Leo, Junxiong Pang, David C. Lye

**Affiliations:** 1 Institute of Infectious Disease and Epidemiology, Tan Tock Seng Hospital, Singapore, Singapore; 2 Department of Clinical Epidemiology, Tan Tock Seng Hospital, Singapore, Singapore; 3 Lee Kong Chian School of Medicine, Nanyang Technological University, Singapore, Singapore; 4 Saw Swee Hock School of Public Health, National University of Singapore, Singapore, Singapore; 5 Yong Loo Lin School of Medicine, National University of Singapore, Singapore, Singapore; University of Malaya, MALAYSIA

## Abstract

**Background:**

Clinically significant bleeding is important for subsequent optimal case management in dengue patients, but most studies have focused on dengue severity as an outcome. Our study objective was to identify differences in admission parameters between patients who developed clinically significant bleeding and those that did not. We sought to develop a model for discriminating between these patients.

**Methods:**

We conducted a retrospective study of 4,383 adults aged >18 years who were hospitalized with dengue infection at Tan Tock Seng Hospital, Singapore from 2005 to 2008. Patients were divided into those with clinically significant bleeding (n = 188), and those without (n = 4,195). Demographic, clinical, and laboratory variables on admission were compared between groups to determine factors associated with clinically significant bleeding during hospitalization.

**Results:**

On admission, female gender (p<0.001); temperature >38°C (p<0.001); nausea/vomiting (p = 0.009) and abdominal pain/tenderness (p = 0.005); lower systolic blood pressure (p<0.001); higher pulse rate (p<0.001); increased absolute neutrophil count (ANC; p<0.001); reduced absolute lymphocyte count (ALC; p<0.001), haematocrit percentage (p<0.001) and platelet count (p = 0.04), and increased prothrombin time (p = 0.003) were significantly associated with clinically significant bleeding on univariate analysis. Multivariate analysis showed that independent variables in the final model were female gender (aOR 2.85; 95% CI: 1.9–4.33); temperature >38°C (aOR 1.81; 95% CI: 1.27–2.61), nausea/vomiting (aOR 1.39; 95% CI: 0.94–2.12), ANC (aOR 1.3; 95% CI: 1.15–1.46), ALC (aOR 0.4; 95% CI: 0.25–0.64), hematocrit percentage (aOR 0.96; 95% CI: 0.92–1.002) and platelet count (aOR 0.993; 95% CI: 0.988–0.998). At the cutoff of -3.919, the model achieved an AUC of 0.758 (sensitivity:0.87, specificity: 0.38, PPV: 0.06, NPV: 0.98).

**Conclusion:**

Clinical risk factors associated with clinically significant bleeding were identified. This model may be useful to complement clinical judgement in triaging adult dengue patients given the dynamic nature of acute dengue, particularly in pre-identifying those less likely to develop clinically significant bleeding.

## Introduction

Acute dengue infection is the most rapidly spreading mosquito-borne viral disease in the world.[1] Dengue is predominantly found in urban and semi-urban areas. The last 50 years has seen a 30-fold increase in its incidence worldwide. Once a disease that affected mainly Asia, Africa, Latin America and the Indian sub-continent, the virus has now spread to the Middle East, Europe, Australia and the USA.[1–7] The World Health Organization (WHO) has reported that over 40% of the global population is at risk of dengue.[8] A 2013 published burden estimate study indicated that there are 390 million infections per year, more than three times the WHO’s estimate.[9]

Over 70% of the worldwide population at risk for dengue lives in Southeast Asian and Western Pacific countries.[1] Previously, dengue in Southeast Asia affected predominantly children, but adults are increasingly being hospitalized with dengue.[10,11]

Dengue has a wide spectrum of clinical presentation. Patients may experience mild symptoms such as abdominal pain, fever or rash in a self-limiting, non-severe clinical course. A small proportion of patients progress to severe disease, which is defined as plasma leakage leading to shock (dengue shock syndrome; DSS) and/or fluid accumulation, with or without respiratory distress; severe bleeding; and/or severe organ impairment.[1] Adverse outcomes also include gastrointestinal bleeding, renal failure, hemoconcentration and abnormalities in hemostasis. Vascular leakage and shock tend to occur more frequently and be more severe in children, while bleeding manifestations and organ involvement are more common amongst adults.[10,12]

Regions of high dengue transmission frequently have seasonal epidemics, which can quickly overwhelm health services. Singapore has experienced several severe dengue outbreaks during the last decade. In 2004, there were 9,459 notified cases, with 83% of these patients being hospitalized.[13] In 2005, 14,209 cases were recorded, which resulted in 25 deaths. Despite continuing efforts to improve dengue surveillance and a world-class vector control program, Singapore experienced another dengue epidemic in 2013, involving 22,170 cases and seven deaths.[14]

The ability to identify patients at high risk of progression to severe bleeding who are likely to benefit from close observation and early intervention with supportive therapy is becoming increasingly important.[15] However, most studies were carried out to identify risk factors for progression focus on general dengue severity [10,12,16–21] or risk factors associated with death.[22–25] Fewer studies were carried out to determine risk factors for any bleeding [26–30]; these studies generally had relatively small patient populations, and the clinical and case definitions varied widely. Even fewer studies were conducted to determine risk factors for severe or clinically significant bleeding.[26,28]

In a study of the clinical characteristics of 6,989 adult patients who were treated for dengue between 2005 and 2008 at a Singapore tertiary hospital, 1,035 (14.8%) had severe dengue (SD) according to WHO 2009 criteria [1]; of these, 40% had severe bleeding.[11] A few cohort studies showed high prevalence of severe bleeding in dengue mortality. A local multi-center study showed elevated risk of death in severe dengue patients.[24] A Taiwanese study showed that massive gastrointestinal bleeding accounted for 40% of dengue hemorrhagic fatalities [25] while a Brazilian study showed 45% of those with gastrointestinal bleeding progressed to death.[31] The aim of this study was to identify simple clinical and laboratory variables that are associated with clinically significant bleeding (CSB) in adult patients who are admitted to hospital with dengue.

## Methods

### Ethics Statement

The study was approved by the National Healthcare Group Domain Specific Review Board (DSRB/E/2008/00567) with a waiver of informed consent for the collection of anonymized data.

### Study Design and Population

This was a retrospective cohort study of all adult patients who were admitted to the Communicable Disease Center at Tan Tock Seng Hospital in Singapore between 1 January 2005 and 31 December 2008 with acute dengue infection. Patients were hospitalized if they were suspected of dengue hemorraghic fever (DHF) or fulfilled the admission criteria as published in another study.[32] Patients were referred to intensive care unit if they had compensated shock (systolic blood pressure > 90 mmHg but narrow pulse pressure < 20 mmHg) and those with hypotension (systolic blood pressure < 90 mmHg). A standardized dengue care path was used to manage patients. On admission, we collected demographic data and information on patients’ past history of fever. Detailed data on clinical signs and/or symptoms, examination findings, microbiological and laboratory parameters were collected daily from hospital admission to discharge. The care path also provided clear criterion for intravenous fluids and blood products. Treatment decision on platelet transfusion was made by individual doctors. Patients without clinical bleeding were often given preventive platelet transfusion if their platelet count was below 20,000. If a patient had clinical bleeding, even though it may not be persistent or severe, often our doctors would administer platelet transfusion. Hospital electronic medical records were used to extract laboratory, microbiological, radiological, treatment and outcome data. Data extraction was performed by medically trained research assistants. More detailed description of the cohort has been described previously.[11] Each patient was assigned a Charlson co-morbidity score if they have any pre-existing illness.[33]

Clinical fluid accumulation was defined as presence of lung dullness or shifiting dullness or pleural effusion or ascites. DHF and DSS were classified as per WHO 1997 definitions.[34]

Dengue patients who were eligible for this study were hospitalized adults aged 18 years or older who either met WHO 1997 or 2009 probable dengue criteria together with positive acute dengue serology (Dengue Duo IgM & IgG Rapid Strip, Panbio Diagnostic, Queensland, Australia),or had a positive RT-PCR result for dengue. Patients who had experienced any bleeding prior to or on the day of admission were excluded from the study.

Clinically significant bleeding (CSB) was defined as any of the following events as evaluated by the clinician: hematemesis, melena, fresh rectal bleeding, menorrhagia, hemoptysis, macroscopic hematuria, intracranial bleeding or a clinical drop in hemoglobin that required whole blood or packed red cell transfusion. These were chosen as they represent clinically significant events to a physician that require closer monitoring and/or further evaluation.

## Statistical Analyses

All variables except the outcomes were evaluated on the day of admission. Variables with less than 10% missing data were imputed with their group medians, or otherwise excluded. Data were randomly split into training (80%) and validation sets (20%). Univariate analysis was performed using the Mann-Whitney test for continuous variables, and the Chi-squared and Fisher’s tests for categorical variables. Variables that had a p-value <0.2 on univariate analysis, or which were considered to be clinically relevant (e.g., age) were subsequently analyzed with multivariate logistic regression to determine independently associated risk factors for CSB in adults. Stepwise regression using Akaike Information Criteria and manual backward elimination methods were carried out for model selection. Sensitivity was the primary measure of performance and was used to choose the final model. Age, gender, illness day on admission and year of admission were added into the model due to potential confounding. The Hosmer-Lemeshow goodness-of-fit test was conducted to ensure that the model fitted the data appropriately. In order to achieve the best discriminatory performance, 0.9 was being used as the minimum sensitivity for the cutoff. All tests were conducted at the 5% level of significance

These analyses were carried out using R software version 3.0.2.[35]

## Results

From 2005 to 2008, 4,383 adults hospitalized for dengue were dengue PCR-positive (n = 1494) or met the WHO criteria for probable dengue with a positive dengue serology (n = 2889). Of these, 188 patients (4.3%) experienced clinically significant bleeding after hospitalization. The breakdown was as follows: menorrhagia (44.7% of patients), hemoptysis (17%), hematemesis (11.2%), macroscopic hematuria (9%), fresh rectal bleeding (9%), melena (8.5%), and decrease in hemoglobin requiring transfusion (5.6%). None of the patients had intracranial bleeding. The proportion of dengue patients who had CSB during each year of the study period ranged from 3.4% in 2005 to 7.4% in 2007. A comparison of demographic features, co-morbidities, symptoms on admission and clinical outcomes among patients with and without CSB are shown in Table 1. Notably, in the cohort, 941 (21.5%) patients had dengue hemorrhagic fever, 158 (3.6%) had dengue shock syndrome, 15 (0.3%) were admitted to intensive care, and 1 died from dengue shock syndrome.

**Table 1:** Demographic and clinical characteristics and outcomes among adult dengue patients with and without CSB.

Variables	CSB (n = 188)	No CSB (n = 4,195)	P-value
**Demographics**			
Age (years)	36 (20–60)	34 (18–60)	0.28^†^
Gender: Female	108 (57.4)	1,351 (32.2)	<0.001^†^
Ethnicity			
Chinese	121 (64.3)	2,906 (69.3)	0.19
Eurasian	0(0)	2(0.1)	NA
Indian	18 (9.6)	478 (11.4)	NA
Malay	16 (8.5)	294 (7)	NA
Others	33 (17.6)	33 (12.2)	NA
**Year of admission**^♀^			
2005	94 (3.4)	2,694 (96.6)	<0.001^†^
2006	22 (6.7)	307 (93.3)	NA
2007	52 (7.4)	649 (92.6)	NA
2008	20 (3.5)	545 (96.5)	NA
**Co-morbidities**			
Diabetes mellitus	10 (5.3)	146 (3.4)	0.22
Hypertension	18 (9.5)	354 (8.4)	0.6
Liver disease	2 (1.06)	30 (0.7)	0.65
Renal disease	2 (1.06)	25 (0.6)	0.32
Charlson’s co-morbidity score >3	1 (0.5)	12 (0.29)	0.43
**Symptoms on admission**			
Temperature > 38°C	117 (62.2)	1,687 (40.2)	<0.001^†^
Illness duration on first visit (days)	5 (3–7)	5 (3–7)	0.001^†^
Myalgia	131 (69.7)	2,853 (68)	0.63
Arthralgia	17 (9)	451 (10.8)	0.45
Headache	90 (47.8)	2,003 (47.7)	0.97
Back pain	18 (9.5)	360 (8.6)	0.6
Chest pain	5 (2.6)	83 (2)	0.42
Eye pain	2 (1.06)	97 (2.3)	0.44
Anorexia	113 (60.1)	2,489 (59.3)	0.83
Rash	63 (33.5)	1,688 (40.2)	0.06^†^
Nausea/vomiting	149 (79.3)	2,985 (70.5)	0.009^†^
Abdominal pain or tenderness	65 (34.5)	1,068 (25.4)	0.005^†^
Clinical fluid accumulation	2 (1)	27 (0.3)	0.36
Narrow pulse pressure	3 (1.5)	21 (0.5)	0.08^†^
Systolic blood pressure < 90 mm Hg	19 (10.1)	217 (5.1)	<0.001^†^
Systolic blood pressure (mm Hg)	103 (82–129)	105 (89–130)	<0.001^†^
Pulse rate (beats per minute)	94 (34–115)	89 (67–122)	<0.001^†^
White cell count (x10^9^/mL)	2.6 (1.3–6.2)	2.7 (1.3–6.5)	0.21
Absolute neutrophil count (ANC; x10^9^/mL)	1.48 (0.49–4.34)	1.4 (0.54–3.62)	0.08^†^
Absolute lymphocyte count (ALC; x10^9^/mL)	0.5 (0.23–1.42)	0.66 (0.26–2)	<0.001^†^
Hematocrit (%)	42.6 (34.4–51.0)	44.5 (36.8–51.2)	<0.001^†^
Creatinine (umol/L)	76 (49–127)	78 (51–112)	0.06^†^
Aspartate transaminase (U/L)	113 (37–785)	113 (35–502)	0.18^†^
Alanine aminotransferase (U/L)	66 (20–591)	69 (20–350)	0.53
Platelets (x10^9^/mL)	57 (18–131)	63 (18–126)	0.04^†^
Prothrombin time (seconds)	13.4 (12.1–16.9)	13.1 (11.9–15.1)	0.003
Partial thromboplastin time (seconds)	33.7 (43–66.9)	41.2 (31.6–56.3)	0.06
**Clinical Outcomes**			
Dengue hemorrhagic fever	112 (59.5)	829 (19.7)	<0.001
Dengue shock syndrome	33 (17.5)	125 (3.0)	<0.001
Platelet transfusion	58 (9.6)	130 (3.4)	<0.001
Blood transfusion	18 (9)	1 (0.02)	<0.001
Hospital length of stay (days)	5 (3–10)	4 (2–7)	<0.001
Intensive care unit admission	8 (4.2)	7 (0.17)	<0.001
Death	3 (1.6)	1 (0.02)	<0.001

Categorical variables are shown as the number and percentage of patients. For continuous variables, the median, 5^th^ and 95^th^ percentile is shown.

^♀^Percentages calculated over each year of admission

^†^Included in the multivariate analysis

There were no statistically significant differences between the groups in terms of co-morbidities. The most commonly reported symptoms on admission were nausea and vomiting, myalgia, anorexia and fever in both groups. Univariate analysis (p<0.05) showed that the following demographic features, clinical symptoms and laboratory results were significantly different on admission among patients who developed CSB compared with those who did not: female gender (p<0.001), temperature >38°C (p<0.001); nausea/vomiting (p = 0.009); abdominal pain or tenderness (p = 0.005), systolic blood pressure less than 90 mmHg (p<0.01), lower systolic blood pressure (p<0.001), higher pulse rate (p<0.001), decreased absolute lymphocyte count (ALC) (p<0.001), reduced hematocrit percentage (p<0.001), reduced platelet count (p = 0.04), and, increased prothrombin time (PT; p = 0.003) (Table 1).

Multivariate analysis showed that the following factors were independently associated with CSB in adults with dengue: female gender, temperature >38°C, increased ANC, decreased ALC and platelet count (Table 2). According to this analysis, women were 2.85 times as likely as men to develop CSB (95% CI: 1.9–4.33).

**Table 2:** Multivariate analysis of clinical factors on admission that were predictive of CSB according to the final model (N = 4383).

Variables	Adjusted OR^†^	(95% CI)
Female*	2.85	(1.9–4.33)
Temperature > 38°C*	1.81	(1.27–2.61)
Nausea/vomiting	1.39	(0.94–2.12)
ANC per 1 unit (x10^9^/mL)*	1.3	(1.15–1.46)
ALC per 1 unit (x10^9^/mL)*	0.4	(0.25–0.64)
Hematocrit per 1 unit (%)	0.96	(0.92–1.002)
Platelet per 1 unit (x10^9^/mL)*	0.993	(0.988–0.998)

^†^Adjusted for age, illness day at presentation and admission year

*Significant at 5% level

For each 0.5-unit increase in baseline ANC, the odds of CSB increased by 1.14 fold (aOR 1.14; 95% CI: 1.07–1.21), and for each 10-unit decrease in baseline platelet count, the odds of CSB increased by 1.07 fold (aOR 1.07; 95% CI: 1.02–1.13).

The stepwise model was deemed superior than the model using manual backward elimination with a higher sensitivity, specificity and model fit. The area under the curve (AUC) of the stepwise model was 0.758, and the Hosmer-Lemeshow goodness-of-fit showed the model fitted the data observed (p = 0.91).

Based on the final model, we created a probability equation with the patients’ relevant clinical data on admission:

Log odds (CSB)=−0.04−(1.05×male gender)+(0.59×temperature>38°C)+(0.3×presence of nausea or vomiting)+(0.27×ANC)+(0.9×ALC)−(0.04×hematocrit percentage)−(0.007*platelet count)

Patients with log odds greater than -3.919 were classified as having a high risk of developing CSB. Using this cutoff of -3.919 from the training data set, the sensitivity for the validation data set was 0.87 and the specificity was 0.38. The positive predictive value (PPV) was 0.06 but the negative predictive value (NPV) was 0.98.

Menorrhagia typically occurs in women of reproductive age, so it was suspected that this could explain the association of female gender with CSB. To test this hypothesis, patients with menorrhagia were excluded from a separate analysis. No female patient had a history abnormal menstrual bleeding. As expected, age and gender were excluded in the model without menorrhagia (Table 3), but the OR for temperature >38°C, baseline ANC, ALC and platelet count remained significant through the model selection process, indicating that these variables were important risk factors for CSB in adult dengue patients, with or without menorrhagia. The model used for this secondary analysis showed an approximately similar discriminatory performance to the model that included menorrhagia (AUC, 0.737; sensitivity, 0.92; specificity, 0.35), which supported the findings that temperature >38°C, baseline ANC, ALC and platelet count were associated with CSB. Aspartate aminotransferase (AST) emerged as a significant predictor after this secondary analysis, even though it was not associated with CSB in the final multivariate analysis (Table 2); however, the adjusted OR for AST was low (1.001; 95% CI:1.000–1.002).

**Table 3:** Secondary multivariate analysis (excluding menorrhagia) of clinical variables predictive of CSB in (n = 4299) adult dengue patients.

Predictors	aOR^†^	(95% CI)
Temperature > 38°C*	1.95	(1.2–3.2)
ANC per 1 unit (x10^9^/mL)*	1.18	(1.04–1.32)
ALC per 1 unit (x10^9^/mL)*	0.3	(0.15–0.57)
Platelet count per 1 unit (x10^9^/mL)	0.995	(0.988–1.001)
AST per 1 unit (U/L)*	1.001	(1.000–1.002)

^†^Adjusted for age, gender, illness day at presentation and admission year

*Significant at 5% level

Females are known to have lower hematocrit values than males. We performed another set of sensitivity analysis stratified by gender. As shown in Table 4, we found that factors associated with clinically significant bleeding were slightly different between male and females. Variables included in the model with males only were temperature > 38°C, systolic blood pressure, hematocrit, ANC, ALC, platelet count, abdominal pain, nausea/vomiting,. For females, variables included were temperature > 38°C, white cell count, ALC, platelet count and abdominal pain.

**Table 4:** Multivariate analysis of clinical variables predictive of CSB in adult dengue patients (stratified by gender).

	Male		Female	
Predictors	aOR^†^	(95% CI)	aOR^†^	(95% CI)
Temperature > 38°C	1.68	(1.2–2.4)*	2.2	(1.3–3.6)*
ALC per 1 unit (x10^9^/mL)	0.45	(0.27–0.70)*	0.36	(0.18–0.72)*
Platelet count per 1 unit (x10^9^/mL)	0.992	(0.987–0.997)*	0.993	(0.985–0.999)*
Abdominal pain	1.35	(0.93–1.94)	1.66	(1.04–2.64)*
Nausea/vomiting	1.6	(1.04–2.5)*	-	-
Systolic blood pressure	0.985	(0.972–0.999)*	-	-
ANC per 1 unit (x10^9^/mL)	1.23	(1.06–1.4)*	-	-
Hematocrit per 1 unit (%)	0.916	(0.883–0.949)*	-	-
White cell count (x10^9^/mL)	-	-	1.2	(0.99–1.43)

^†^Adjusted for age, illness day at presentation and admission year;

*Significant at 5% level

## Discussion

The annual proportion of adult dengue patients admitted to our hospital during the study period who developed CSB ranged from 3.4% to 7.4%. Knowledge of risk factors of CSB in adult dengue patients can assist with preparedness, improve patient management, and facilitate the appropriate use of limited resources.

This large retrospective study in adult patients hospitalized with dengue over a 4-year period showed that female gender, temperature >38°C, nausea/vomiting, increased ANC, and lowered ALC, hematocrit percentage and platelet counts were associated with CSB by multivariate analysis. Although nausea/vomiting and hematocrit percentage were not statistically significant with odds ratios crossing 1, they were still clinically important. Of the 188 adult dengue patients who had CSB, 44.5% had menorrhagia, which may explain the increased effects of female gender on multivariate analysis. Once patients with menorrhagia were excluded from the analysis, temperature >38°C, increased ANC and reduced ALC and platelet count remained as significant variables of CSB. AST appeared statistically significant in the secondary multivariate analysis which excluded menorrhagia, but the OR was very small. The fact that it was not revealed by the final multivariate analysis suggested a weak association in our patient population, particularly as the OR was small and the patient numbers were reduced. Another possible explanation is that AST was a risk factor of CSB in adult dengue patients without menorrhagia.

We also found that clinically significant bleeding in males and females was associated with different risk factors. There were, however, some variables that remained significant in both males and females, namely abdominal pain, platelet count, ALC and temperature>38°C. The latter three variables were significant in all four multivariable models.

Our findings are in agreement with those from several other studies. In a study of early predictors of clinically significant bleeding amongst 277 patients with dengue aged >15 years, multivariate analysis showed female gender (OR, 14.52; 95% CI: 0.16–0.56, p<0.001), absolute lymphocyte count >550/μl (OR 5.78; 95% CI: 1.17–4.99, p = 0.02) and severe thrombocytopenia (OR 4.72; 95% CI: 0.13–0.9, p = 0.03) to be independently related to clinically significant bleeding.[26] A case-control study of 59 dengue patients aged >17 years with or without bleeding showed that thrombocytopenia (OR 200; 95%CI: 22–1000; p = 0.0003), monocytosis (OR 13.5; 95% CI: 3,0–48,6; P < 0.0001) and elevated D-dimer (OR 14.9; 95% CI: 2.4–90; p = 0.0032) were the parameters most strongly associated with bleeding complications; ROC analysis showed that platelets < 67,000/mm^3^ (P < 0.0001, 94% sensitivity and 93% specificity) and monocytes > 715/mm^3^ (P < 0.0001, 84% sensitivity and 82% specificity) predicted dengue patients with bleeding.[29] In a large prospective cohort study of 643 patients aged 5 years or older who had acute febrile syndrome including dengue, the factors predictive of spontaneous non-cutaneous bleeding were age between 12 and 45 years (RR 2.22, 95% CI: 1.25–3.94), rash (RR 1.66; 95% CI: 1.25–2.2), vomiting (RR 1.46; 95% CI: 1.16–1.83), temperature >38°C (RR 2.63; 95% CI: 1.6–4.33), leukocyte count <4,500/μL (RR 1.87; 95% CI: 1.19–2.96), and platelet count <90,000/μL (RR 1.8; 95% CI: 1.1–2.94).[27] In a study of 132 children with or without spontaneous skin or mucosal bleeds, predictive factors for spontaneous bleeding derived from logistic regression analysis were platelet counts <50x10^9^/L, ALT more than 3 times the normal value, a biphasic pattern of fever, and abdominal pain.[30]

Factors other than those identified in our study, namely abdominal pain, hepatomegaly, pleural effusion and elevated liver enzymes [7,36], were associated with bleeding in other studies[7,28,36–38], but were not conclusively significant in ours. However, there were inconsistencies in the literature as to whether elevated liver transaminases were associated with dengue severity and with bleeding. A previous study in dengue patients at our center between 2006 and 2008 showed that, although aminotransferase levels did increase in conjunction with dengue severity, AST or ALT values did not discriminate between DF and DHF or non-severe and severe dengue.[39] AST and ALT levels were elevated in patients with DHF compared with those with DF in another South American study of patients aged >5 years, but they were not independent predictors of severity.[40] A prospective cohort study of 203 DF patients in South America showed that AST levels were higher in patients with DHF than those without, but that AST was not an independent predictor of disease severity in patients with DF.[40] However, a meta-analysis has shown a strong association between elevated AST and ALT and dengue shock syndrome (DSS).[18] Elevated AST/ALT levels were associated with clinically significant bleeding after univariate analysis in the Thai study mentioned above, but not after multivariate analysis.[26] A prospective study on Vietnamese adults identified vomiting ≥3 times a day, lymphopenia, thrombocytopenia and elevated liver enzymes to be associated with plasma leakage, while elevated alanine aminotransferase with internal bleeding.[16] A retrospective study of Singaporean adults showed that clinical bleeding, lymphopenia, elevated serum urea and low serum protein to be associated with dengue hemorrhagic fever.[13]

Although our model managed to predict 86% of actual cases correctly, our cutoff also misclassified many non-CSB patients. The PPV reduces as the prevalence falls. The prevalence of CSB in our cohort was approximately 4%, which made achieving a high PPV difficult since the chance of developing CSB was low to begin with.[41] The high NPV of 0.98 however may be useful for ruling out patients without CSB on admission. For example, a female patient admitted without nausea, fever and average ANC, ALC, hematocrit and platelet based on our cohort in Table 1 has a 9% chance of developing CSB.

Our retrospective study has some limitations. Firstly, it involved patients admitted at a single center. However, it included a range of ethnic groups that are fairly representative of the population of Southeast Asia. Secondly, we used laboratory values taken only on the day of admission, and dengue is a dynamic disease that can change from hour to hour. Dengue patients also tended to be admitted to our center at a later stage of disease [median day 5 (range, days 3–7) of illness in both groups] because it is a tertiary hospital, hence our findings may not be applicable in the earlier phase of dengue. However, given that most abnormal laboratory values observed over the course of dengue illness are seen during days 3–6 or even days 7–10 after the onset of illness [7,36,38,42], it is likely that, even though we only obtained measurement of these variables on the day of admission, the day of illness on which they were assessed would generally have been close to, the peak or nadir of abnormality. Thirdly, external validity may be limited as our adult cohort cannot be generalized to children. Further validation from other adult populations is needed as we could only split our data into one validation set. Lastly, the definition of menorrhagia in this paper was patient reported and classified by the doctor if patient had excessive amount of clots and number of pads than usual. It is difficult to measure the volume of menstrual bleeding clinically. There may be possible response bias but we felt that this was pragmatic for our circumstance. In addition, performing sensitivity analysis without menorrhagia reduced the number of cases substantially.

The strengths of our study are that it involved a large patient population that was admitted to our center over a relatively long period of time, during which the number of annual cases varied widely following multiple outbreaks, and the predominant serotype changed from dengue DENV1 to DENV2 in 2007.[43]

In summary, we have identified several relevant clinical and laboratory markers that are associated with CSB in adult patients admitted to hospital for dengue which are particularly useful for ruling out patients with CSB. Healthcare personnel can view patients as safe from CSB when one or more of the following risk factors are absent: female gender, temperature >38°C, nausea/vomiting, increased neutrophil count, and decreased lymphocyte count, hematocrit and platelet counts. These findings, complemented with clinical judgement in reviewing new daily clinical and laboratory data given the dynamic nature of dengue disease progression, may provide an indication of patients that are safe not to admit to the hospital.

## Supporting Information

S1 Checklist STROBE checklist for cohort studies. (DOC)
